# Introducing CCD1 into isolated *Rhodotorula* strain enhances flavor production and improves cigar fermentation

**DOI:** 10.3389/fbioe.2024.1510075

**Published:** 2024-12-03

**Authors:** Sida Guo, Yasen Li, Beibei Zhu, Qianying Zhang, Zhen Yang, Yun Jia, Quanwei Zhou, Zhengcheng Zhang, Dongliang Li

**Affiliations:** ^1^ Cigar Fermentation Technology Key Laboratory of China Tobacco, China Tobacco Sichuan Industrial Co., Ltd., Chengdu, Sichuan, China; ^2^ Industry Efficient Utilization of Domestic Cigar Tobacco Key Laboratory of Sichuan Province, China Tobacco Sichuan Industrial Co., Ltd., Shifang, Sichuan, China

**Keywords:** cigar fermentation, *Rhodotorula mucilaginosa*, CCD1, carotenoids, flavor components

## Abstract

**Introduction:**

The fermentation process plays an important role in enhancing the quality of cigar tobacco leaves. Through fermentation, microbial metabolism can degrade aromatic precursors and macromolecules, which increases the content of aroma compounds and reduces irritancy of tobacco leaves.

**Methods:**

To further enhance the fermentation effect of cigar tobacco leaves, a Rhodotorula strain (Rh3), capable of producing carotenoids and improving fermentation quality, was isolated from cigar tobacco leaves. Subsequent genetic engineering techniques introduced the carotenoid cleavage dioxygenase 1 (CCD1) gene into the isolated Rh3.

**Results:**

The modified Rh3 exhibits a significant increase in carotenoid degradation products compared with the original Rh3 in culture medium (from 0.29 μg/mg to 15 μg/mg). Subsequent cigar tobacco leaf fermentation experiments revealed that the modified Rh3 produced 65.9% more carotenoid degradation products compared to the control group, outperforming the original strain, which achieved a 41.4% increase. Furthermore, the modified strain preserves its ability to improve the intrinsic chemical composition of cigar tobacco leaves.

**Discussion:**

We show here that modified Rh3 can increase the content of carotenoid degradation products, thereby enhancing the fermentation effect of cigar tobacco leaves. This study presents a beneficial exploration to improve the quality of cigar tobacco leaves for future use and offers a promising strategy for producing flavor compounds from discarded tobacco leaves.

## 1 Introduction

Cigars are a specialized type of tobacco product rolled from dried and fermented cigar tobacco leaves (CTLs). Fermentation process plays a crucial role in converting aromatic precursors and degrading macromolecules in CTLs. Traditional spontaneous fermentation only relies on the microorganisms naturally present in tobacco leaves and surrounding environment. When spontaneous fermentation fails to meet the required quality, exogenous microorganisms are introduced to enhance fermentation effects. These include *Acinetobacter*, which produces aldehyde and ketone compounds (Zheng et al., 2022); *Candida*, which degrades nicotine (Jia et al., 2023b); *Bacillus*, which secretes various enzymes (Ma et al., 2024); and certain microorganisms that degrade carotenoids to produce various aroma compounds (Wu et al., 2023b). After fermentation, the intrinsic chemical composition, flavor component content and smoking quality of CTLs can be significantly improved. The content of flavor components in CTLs is one of the crucial factors influencing cigar quality and serves as the material basis of the tobacco aroma style (Liu et al., 2022). Most flavor components are converted from several aromatic precursors, including carotenoids, chlorophyll, cembranoids, and phenylpropanoids (Jia et al., 2023b). Through fermentation and aging, carotenoids can be degraded into a variety of flavor compounds such as ionone, dihydroactinidiolide, and megastigmatrienone (Jia et al., 2023a). Research has shown that spraying carotenoids to tobacco leaves before fermentation enhances the content of aromatic compounds, significantly improves aroma quality while reducing irritability (Liu et al., 2010). The above studies indicate that carotenoids, especially their degradation, play an important role in CTL fermentation process.

*Rhodotorula* strains have the ability to utilize a wide range of carbon and nitrogen sources (Ghilardi et al., 2020). It exhibits strong environmental adaptability and has become a promising host for fermentation and carotenoid production (Dias Rodrigues et al., 2019). Studies have demonstrated that *R. mucilaginosa* can be utilized in soy protein solid-state fermentation to produce plant-based meat analogues (Ou et al., 2023), converting depolymerized sugars and aromatics in engineered feedstocks to bisabolene (Rodriguez et al., 2019), enhancing aroma compounds in Ecolly dry white wine (Wang et al., 2017), and utilizing food wastes to generate carotenoids (Liu C. et al., 2021). Furthermore, carotenoids produced by *R. mucilaginosa* can be used not only as ingredients for food, feed, and pharmaceutical products, but also as crucial precursors of flavor compounds in CTLs. These findings suggest that *Rhodotorula* possesses the potential to improve the CTL fermentation process.

Carotenoids produced by *Rhodotorula* are encapsulated within fungal cells (Allahkarami et al., 2021), which limits their accessibility to microorganisms and enzymes present in CTLs during tobacco fermentation. This cellular compartmentalization impedes the complete conversion of these pigments into aroma compounds. CCD1, a key enzyme in carotenoid metabolism, catalyzes the oxidative cleavage of various carotenoids at the 9, 10 (9′, 10′) double bond position (Zhou et al., 2019). This cleavage produces crucial aroma constituents, including ionones, geranylacetone, and damascenone, which significantly contribute to the complex flavor profiles and overall quality of cigars (Wu et al., 2023). Recent studies have successfully cloned and expressed plant-derived CCD1 genes in yeast species, such as *Saccharomyces cerevisiae* (Timmins et al., 2023) and *Yarrowia lipolytica* (Czajka et al., 2018), to generate apocarotenoid aromatic compounds.

Previous studies on CTL fermentation primarily focused on isolating microorganisms from CTLs and reintroducing them into the fermentation process to accelerate CTL biomass conversion. However, studies on modifying isolated microorganisms to enhance their fermentation capabilities remain limited. To facilitate the degradation of *Rhodotorula* intracellular carotenoids and generate beneficial degradation products, our study utilized genetic engineering technique to introduce CCD1 gene into the isolated Rh3 strain. This modification intends to further improve the fermentation effects of the Rh3 strain. To our knowledge, this is the first report of modifying microorganisms isolated from CTLs with the aim of increasing the carotenoids degradation product content. Our study presents a valuable exploration to elevate CTL quality for future applications and offers a promising strategy for producing flavor compounds from discarded tobacco leaves.

## 2 Material and methods

### 2.1 Materials and reagents

Reagents used in this study were purchased from TIANGEN Technology Co., Ltd., (Beijing, China), unless noted otherwise. Potato Dextrose Agar (PDA), Potato Dextrose Broth (PDB), Lysogeny Broth (LB) medium, Ampicillin sodium, Hygromycin B, Cefotaxim sodium salt, and Acetosyringone were purchased from Solarbio Technology Co., Ltd., (Beijing, China). 2-Morpholinoethanesulphonic acid (MES), FeSO_4_·7H_2_O, and (NH_4_)_2_SO_4_ were purchased from Aladdin Technology Co., Ltd., (Shanghai, China). Fungal genomic DNA extraction kit was purchased from Biospin Co., Ltd., (Hangzhou, China). QuEChERS extraction kit, QuEChERS SPE kit, and other materials required for GC-MS were purchased from Agilent (Santa Clara, CA, United States). The cigar tobacco leaves (DX1), harvested in 2021 from Sichuan Province, China, were collected and provided by China Tobacco Sichuan Industrial Co., Ltd. (Sichuan, China).

### 2.2 Isolation of *Rhodotorula* strains

To isolate pigment-producing strains from CTLs, 5 g CTLs were added to 200 mL sterilized PBS solution (pH 7.2) and shaken at 180 rpm, 30°C for 2 h. The mixture was filtered, and the suspension was then spread on PDA plates (with ampicillin sodium). Microorganisms forming red colonies were isolated and cultured. Genomic DNA was extracted and amplified with ITS1 (5′-TCC​GTA​GGT​GAA​CCT​GCG​G-3′) and ITS4 (5′-TCC​TCC​GCT​TAT​TGA​TAT​GC-3′). The PCR products were sequenced by Sangon Biotech (Shanghai, China), followed by comparing with the identified species using BLAST (Basic Local Alignment Search Tool). After identification, the isolated Rh3 strain was deposited in the China General Microbiological Culture Collection Center (CGMCC), Beijing, China, under accession number CGMCC 30933. The ITS sequence of Rh3 was submitted to the NCBI GenBank (PP956787).

### 2.3 Cigar tobacco leaf fermentation

*Rhodotorula* strains were individually cultured in potato dextrose water medium for 48 h at 28°C 200 rpm. Cells were harvested by 900 × *g* centrifugation for 10 min at 4°C. The harvested cells were then inoculated into 5 kg CTLs with 30% water content at a cell density of 1 × 10^6^ CFU/g. CTLs with the same water content but without inoculation were used as control. All samples were subsequently fermented at 30°C (70% humidity) for 28 days. After fermentation, samples were prepared for further analysis.

### 2.4 Sensory quality evaluation

After fermentation, the CTLs were rolled into cigars (110 mm length, 14 mm diameter). The cigars were then maintained at 20°C and 60% relative humidity to equilibrate their water content. The sensory quality was evaluated following the Standard Evaluation Form provided by Great Wall Cigar Factory (Jia et al., 2023b). Ten well-trained assessors, specializing in cigar production and evaluation, participated in sensory quality evaluation. For quality characteristic, twelve parameters, such as richness, matureness, irritation, etc., were evaluated using a 10-point scale (0–9). Higher scores indicate better performance for each characteristic. Flavor characteristics, such as bean, baking, nutty, etc., were evaluated using a 5-point scale (1–5). Higher scores indicate stronger flavor intensities. The evaluation score of each sample was consistently recognized by all panelists.

### 2.5 Construction of CCD1-expressing Rh3 strain

The codon-optimized *Osmanthus fragrans* CCD1 gene (Supplementary Material S1) was synthesized by Sangon Biotech (Shanghai, China). The optimized OfCCD1 gene was amplified with OfCCD1-F (5′- TCA​CAG​ATA​TCG​CCA​CCA​TGC​ACC​ACC​ACC​ACC​ATC​ACG​GCA​TGC​AGG​GCG-3′) and OfCCD1-R (5′-TCG​TAC​TAG​TTC​AGA​CCT​TCG​CCT​GCT​CCT-3′). The PCR product was digested with EcoRV/SpeI and ligated into the pZPK-MCS plasmid (kindly provided by Prof. Zongbao K. Zhao of Dalian Institute of Chemical Physics, Chinese Academy of Sciences), (Sun et al., 2017). The constructed pZPK-OfCCD1 plasmid was transformed into *A. tumefaciens* AGL1 and subsequently transformed into Rh3 via Agrobacterium-mediated transformation (ATMT) (Lin et al., 2014). Hygromycin B resistant colonies were selected. The successful transformation was confirmed by PCR-based genotyping and Sanger sequencing of the genomic DNA.

### 2.6 Characterization of modified Rh3 strain

Verification of CCD1 integration by agarose gel electrophoresis: After genomic DNA extraction, the genomic DNAs were subjected to PCR amplification and agarose gel electrophoresis (1% agarose gel, 120 V for 30 min) to confirm presence of CCD1 gene. Colony morphology: After streaked on an PDA plate and cultured at 28°C for 48 h, the colony color of original and modified Rh3 were observed. Pigment extraction and analysis: Cells were harvested by centrifugation and washed 2–3 times with PBS. The cell pellets were thoroughly resuspended in 0.7 mL DMSO and incubated at 55°C for 10 min. Subsequently, 0.7 mL acetone was added and mixed thoroughly, followed by incubating at 45°C for 15 min. After centrifugation at 13,800 × *g* for 5 min, the supernatant containing the extracted carotenoids was collected and analyzed by spectrophotometer. Growth curve analysis: Pre-cultured Rh3 strains were inoculated into 50 mL PDB medium and incubated at 28°C, 200 rpm. Growth was monitored by measuring OD600.

### 2.7 Analysis of flavor components produced in original and modified Rh3 strains using headspace SPME GC-MS

Flavor components in both the original and modified Rh3 samples were analyzed in triplicate using headspace solid-phase microextraction GC-MS (HS-SPME-GC-MS) (Grondin et al., 2015; Slaghenaufi and Ugliano, 2018; Hu et al., 2023). After 48 h of cultivation in PDB medium, samples (0.1 g) of both strains were harvested and transferred to headspace vials. SPME analysis was performed using 50/30 μm DVB/CAR/PDMS fiber. 50 μL phenylethyl acetate (10.477 mg/mL) was utilized as internal standard. For experimental setup, samples were incubated at 80°C for 30 min, followed by extraction at 80°C for 40 min. Thermal desorption was conducted at 250°C for 5 min. Subsequently GC-MS analysis was performed by Agilent 8890/5977B GC-MSD with DB-5MS column (60 m × 1.0 μm × 0.25 mm) under the following conditions: 1 mL/min helium (purity 99.999%, *v*/*v*) was used as carrier gas. The temperature program was initiated at 60°C, with a ramping rate of 5°C/min until reaching 280°C, and held at 280°C for 10 min. The mass spectrometer was operated at an ionization energy of 70 eV in full scan mode, with a mass range of 31–550 atomic mass units. Compound identification was performed using the NIST17 spectral library.

### 2.8 Chemical composition analysis of fermented cigar tobacco leaves

After fermentation by the original and modified Rh3 strains, the flavor components of CTLs were analyzed in triplicate using untargeted metabolomics techniques via gas chromatography-mass spectrometry (Agilent 8890/5977B GC-MSD), following previously established protocols (Hu et al., 2022a; Jia et al., 2023b; Wu et al., 2021). Metabolite extraction was performed using a QuEChERS extraction kit. CTL powder (2 g) was thoroughly infiltrated with 10 mL deionized water, followed by the addition of 10 mL acetonitrile and 50 μL phenylethyl acetate (internal standard, 10.477 mg/mL). The mixture was shaken at 2,000 rpm for 2 h and subsequently frozen at −20°C for 10 min. A salt mixture containing 4 g MgSO_4_, 1 g NaCl, 1 g NaCitrate, and 0.5 g disodium citrate sesquihydrate was added, followed by immediate shaking for water removal. Subsequently, 1 mL supernatant was mixed with 0.15 g MgSO_4_ and shaken at 2,000 rpm for 2 min. After centrifugation at 3,500 × *g* for 2 min, the supernatant was collected for GC-MS analysis. Metabolite separation was performed on a DB-5MS column (60 m × 1.0 μm × 0.25 mm). The GC oven temperature program was initiated at 60°C, ramped to 250°C at a rate of 2°C/min, then to 290°C at a rate of 5°C/min, with a final 20-min hold. The MS was operated at an ionization voltage of 70 eV with a mass scan range of 26–400 amu.

The major chemical components of CTLs, including total alkaloids, total sugar, reducing sugar, total nitrogen, potassium, and chlorine, were analyzed in triplicate by continuous flow analytical system, following the method described by Liu F. et al. (2021).

### 2.9 Statistical analysis

Heatmaps were generated using the pheatmap package in R. Statistical analyses were performed using SPSS (Version 26.0). Further data analyses and graph plotting were conducted using GraphPad Prism 8.

## 3 Results

### 3.1 Isolation of *Rhodotorula* strains

**FIGURE 1:**
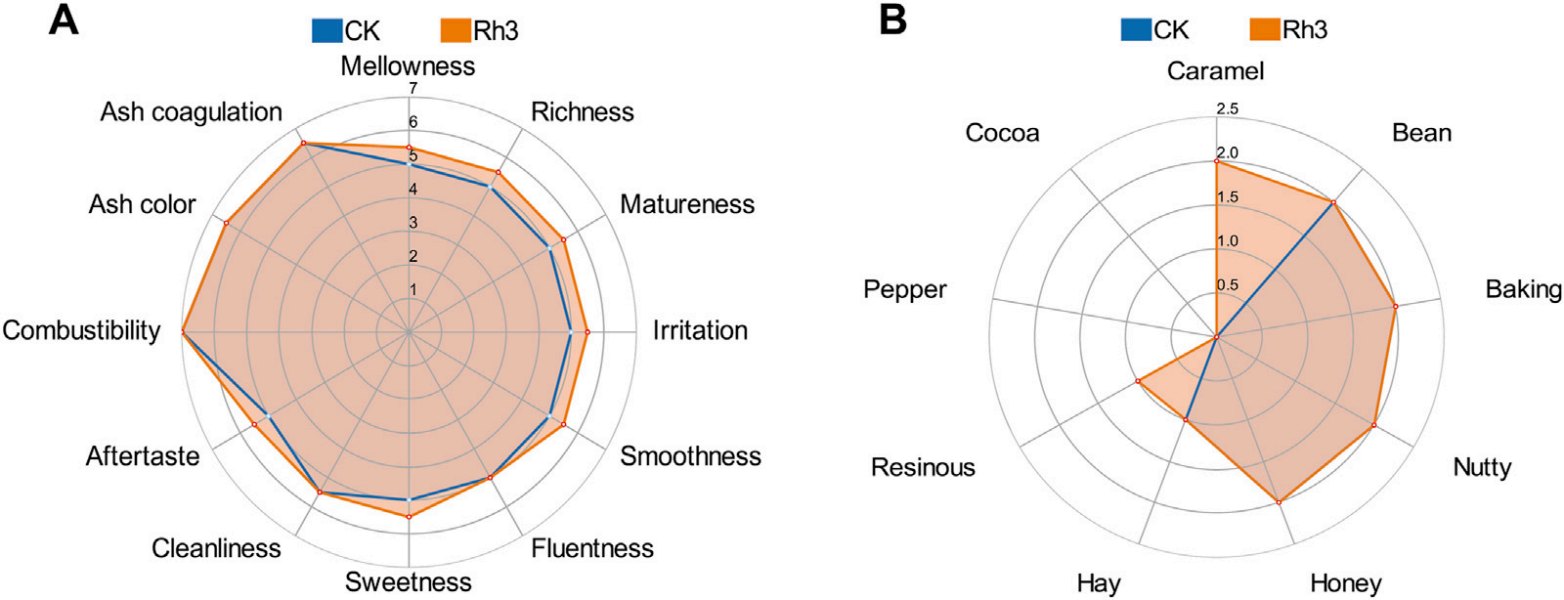
Sensory evaluation radar plots of CTLs fermented by Rh3 and water. **(A)** Quality characteristics of different fermentation groups. **(B)** Flavor characteristics of different fermentation groups. CK represent control group.

*Rhodotorula* strains exhibit strong environmental adaptability and can produce various aromatic compounds (Wang et al., 2024) and carotenoids which are crucial precursors of flavor components in cigars. Therefore, three microbial strains with varying pigment-producing capacities were isolated from CTLs (Supplementary Figure S1). Subsequent ITS sequencing revealed that all three isolated strains exhibited over 99% sequence identity with *Rhodotorula mucilaginosa*. Notably, strain Rh3, which demonstrated the highest carotenoid yield and CTL fermentation effect, displayed 99.65% sequence homology to the *R. mucilaginosa* reference strain. Sensory quality evaluation showed that the isolated Rh3 could reduce irritancy, enhance maturity, aroma intensity, and smoothness of CTLs through fermentation (Figure 1A). Furthermore, CTLs fermented by strain Rh3 showed increases in caramel sweet and resinous flavor (Figure 1B). These findings indicate that isolated Rh3 can be effectively utilized in CTL fermentation.

### 3.2 Integrating CCD1 gene into Rh3 genome

To further improve the fermentation efficiency of strain Rh3, this study aimed to degrade carotenoids in strain Rh3 to produce more flavor components during fermentation. However, the carotenoids synthesized by Rh3 are encapsulated within the fungal cells, making it challenging for exogenous microorganisms or enzymes to completely degrade them into aroma compounds. Since CCD1 gene has been widely utilized to cleave carotenoids and generate carotenoid degradation products (Czajka et al., 2018; Floss and Walter, 2009; Gong et al., 2022; Timmins et al., 2023), which are crucial for improving the quality of cigars. Therefore, we intended to integrate CCD1 gene into the genome of strain Rh3, utilizing genetic engineering technique to further enhance the fermentation efficiency of strain Rh3.

**FIGURE 2:**
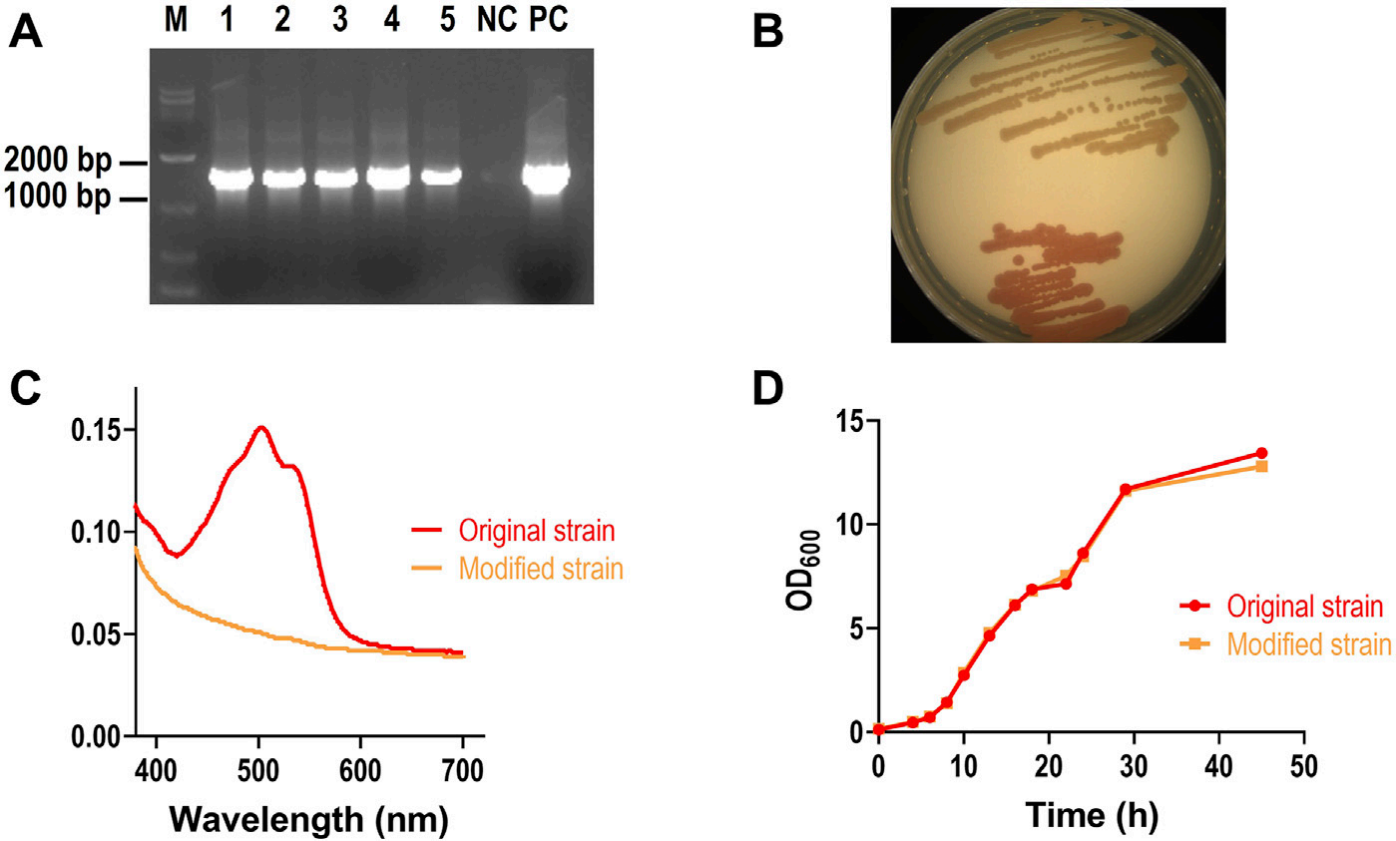
Characterization of modified Rh3 strain. **(A)** PCR verification of CCD1 integration into Rh3 genome. Lanes 1–5: five modified Rh3 genomes, NC: original Rh3 genome, PC: pZPK- CCD1 plasmid. **(B)** phenotype confirmation of modified Rh3 strain (Upper) and original Rh3 strain(lower). **(C)** Absorbance spectrum of pigment extracted from modified Rh3 strain and original Rh3 strain. **(D)** Growth curves of modified strain and original strain.

After transformation, genomic DNA of original and modified Rh3 strains were extracted. PCR amplification targeting the CCD1 gene showed successful amplification only in modified Rh3 strains (Figure 2A), indicating successful gene integration. Additionally, the change in colony color from red to orange (Figure 2B) and the decrease of pigment absorbance in modified strain (Figure 2C) demonstrated that red carotenoids accumulated by strain Rh3 were degraded by CCD1, which confirmed successful CCD1 expression. To determine whether CCD1 expression affects the growth of strain Rh3, the growth curves of both the original and modified strains were analyzed. The result showed that two growth curves are basically overlapped, suggesting that CCD1 expression does not affect the growth of strain Rh3 (Figure 2D).

### 3.3 Detecting carotenoid degradation products in CCD1 expressing Rh3 strain

**FIGURE 3:**
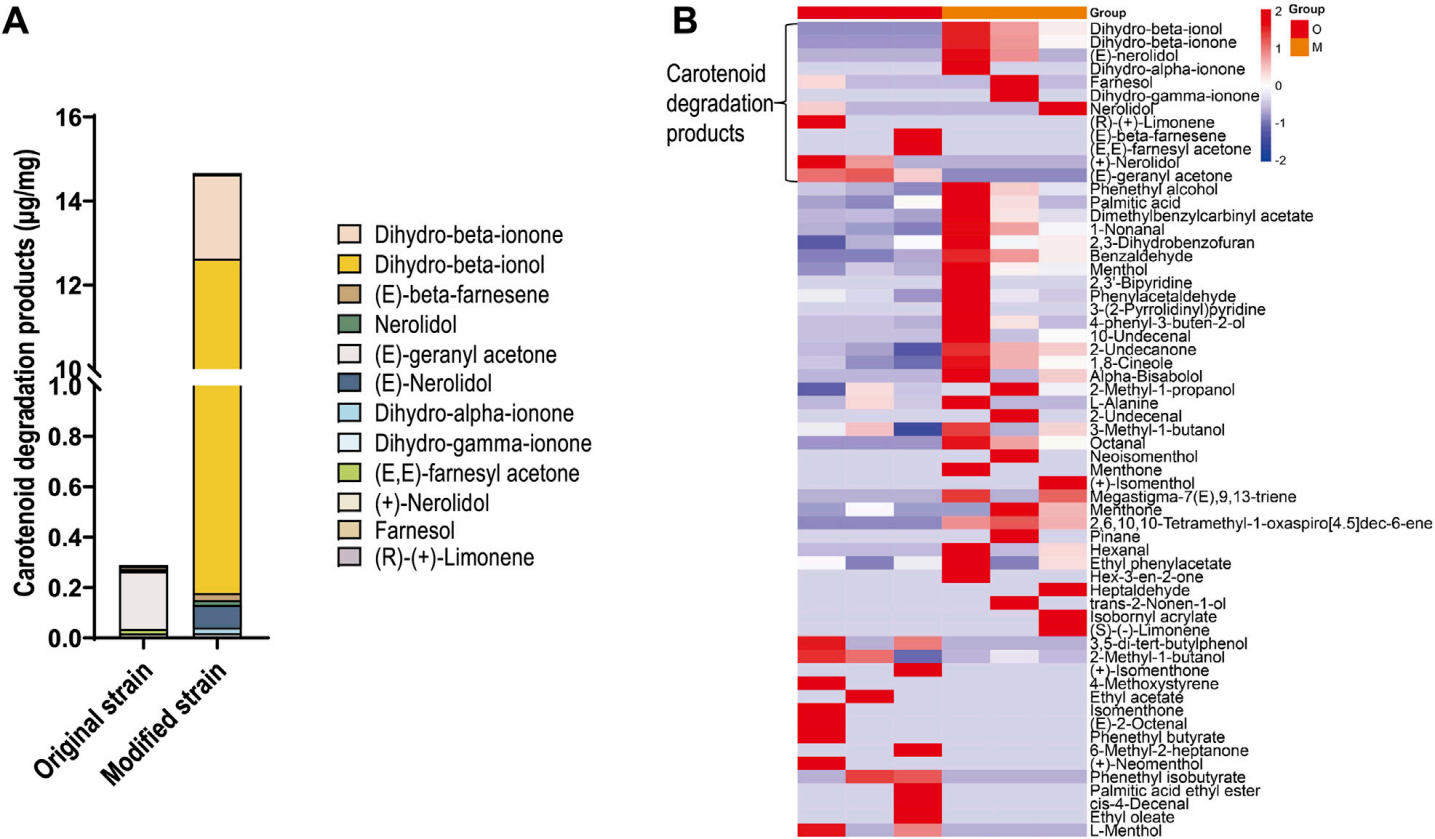
Differences of flavor component produced by original and modified Rh3 strains **(A)** Carotenoid degradation products content in original and modified Rh3 strains. Data represents an average of triplicate experiments. **(B)** Heatmap of changes in flavor components between original and modified Rh3, experiments were performed in triplicate. O represents original Rh3 strain, M represents modified Rh3 strain.

Carotenoid degradation products represent one of the most important classes of flavor compounds in cigars. To investigate whether CCD1 expression in strain Rh3 catalyzes carotenoid degradation and produces carotenoid degradation products, SPME-GC-MS was conducted to detect metabolites in both the original and modified Rh3 strains. The results revealed that the original strain produced minimal amounts of carotenoid degradation products, whereas the modified strain exhibited a significant increase in these compounds (Figure 3A), with dihydro-beta-ionol and dihydro-beta-ionone being the predominant components.

In detail, compared with the original strain, seven carotenoid degradation products (especially dihydro-beta-ionone, dihydro-beta-ionol, and (E)-nerolidol) were found in higher concentrations in the modified strain (Figure 3B). Five carotenoid degradation products, including dihydro-beta-ionone, (E)-nerolidol, and dihydro-gamma-ionone were exclusively detected in the modified strain. Furthermore, similar to the original strain, the modified strain could also produce various volatile flavor compounds that enhance tobacco flavor, such as phenethyl alcohol and dimethyl benzyl carbinyl acetate. These findings indicate that CCD1 expression in strain Rh3 facilitates carotenoid cleavage and generates flavor compounds that are important for improving cigar quality.

### 3.4 Comparative analysis of flavor components between the original and modified Rh3 in cigar tobacco leaf fermentation

To further confirm that CCD1 expression in the Rh3 strain benefits cigar fermentation, both the original and modified Rh3 strains were separately inoculated to ferment CTLs. CTLs with the same water content but without microorganism inoculation were served as control.

**FIGURE 4:**
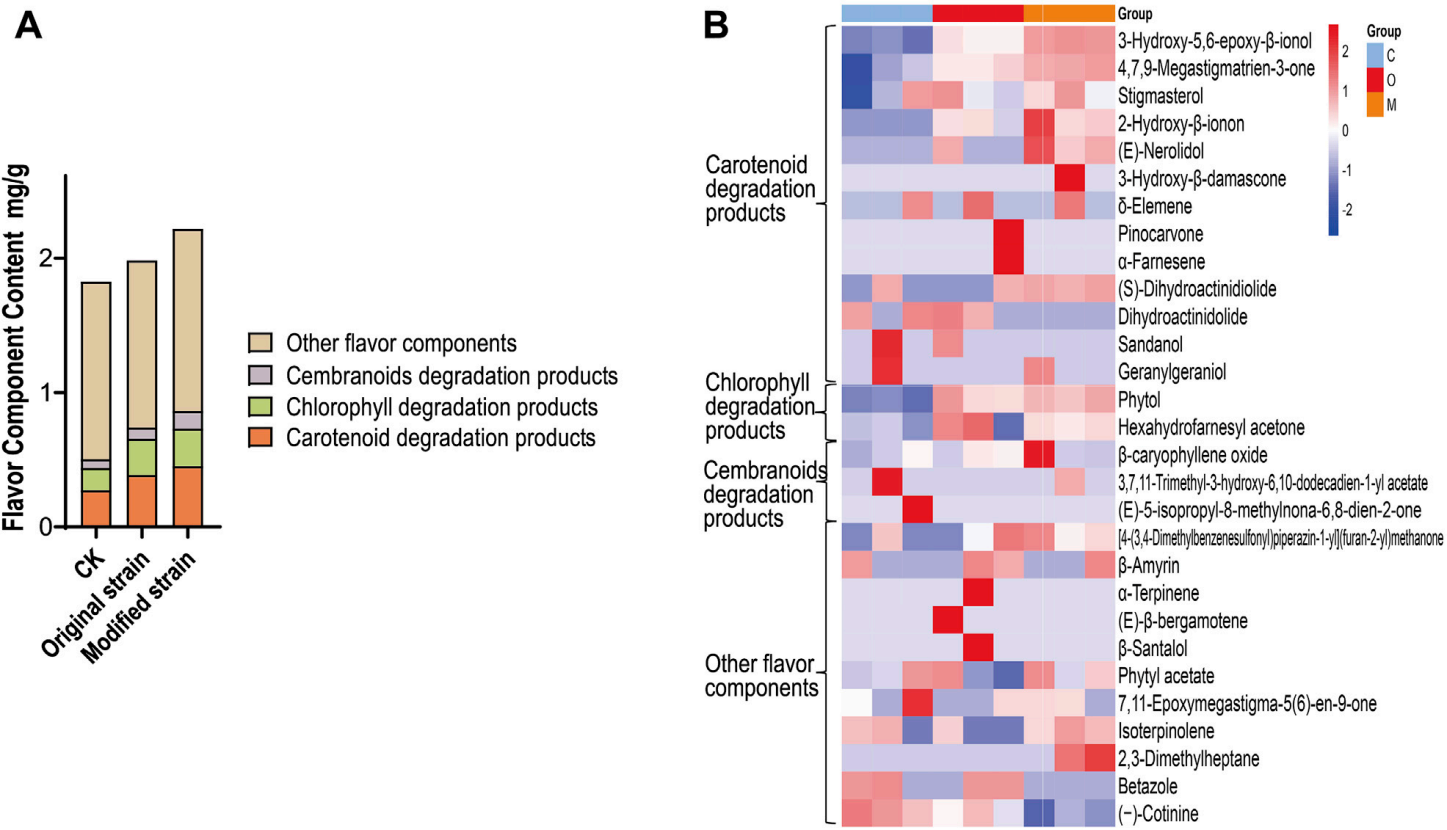
Differences of flavor components in CTLs after original and modified Rh3 fermentation. **(A)** Flavor component content of CTLs in different fermentation groups (classified by precursors). CK represents control group. **(B)** Heatmap of flavor components in CTLs of different fermentation groups, experiments were performed in triplicate. C represents control group, O represents original strain fermentation group, M represents modified strain fermentation group.

The content of flavor components is a reliable indicator of cigar quality (Hu et al., 2023; Jia et al., 2023b). GC-MS analysis was performed to determine the flavor component content in cigar leaves. Based on their precursors (Leffingwell, 2002; Yan et al., 2016), the detected flavor components were categorized into four categories: carotenoid degradation products, chlorophyll degradation products, cembranoid degradation products, and other flavor components (Figure 4A). As expected, compared with the control group, the original strain group exhibited a 41.4% increase in carotenoid degradation products, while the modified strain group showed a 65.9% increase. This result proves that the modified Rh3 can further enhance the yield of carotenoid degradation products in fermented tobacco leaves on the basis of original Rh3.

Chlorophyll degradation products also significantly contribute to the sensory profile of cigars. In both the original and modified strain fermentation groups, the content of chlorophyll degradation products was approximately 0.27 mg/g, which is higher than that observed in the control group. This result indicates that the Rh3 strain possesses the ability to enhance the chlorophyll degradation products through CTL fermentation, and the introduction of CCD1 does not adversely affect chlorophyll degradation. The total content of flavor components increased from 1.82 mg/g in the control group to 1.98 mg/g with original Rh3 fermentation, and further increased to 2.22 mg/g with modified Rh3 fermentation. These findings demonstrates that the modified Rh3 strain generates more flavor components during CTL fermentation, thereby enhancing the fermentation efficiency.

The changes in flavor component profiles among different fermentation groups are shown in Figure 4B. Most of the detected carotenoid degradation products, including 3-hydroxy-5,6-epoxy-β-ionol (Baldermann et al., 2005) and 4,7,9-megastigmatrien-3-one (Yao et al., 2024), showed higher content in both original and modified Rh3 strain fermentation groups compared to the control group. Notably, the modified group exhibited the highest levels. These results align with our hypothesis that introducing CCD1 into Rh3 enhances the generation of carotenoid degradation products, which are crucial for improving cigar quality.

### 3.5 Comparative analysis of major chemical compositions

**FIGURE 5:**
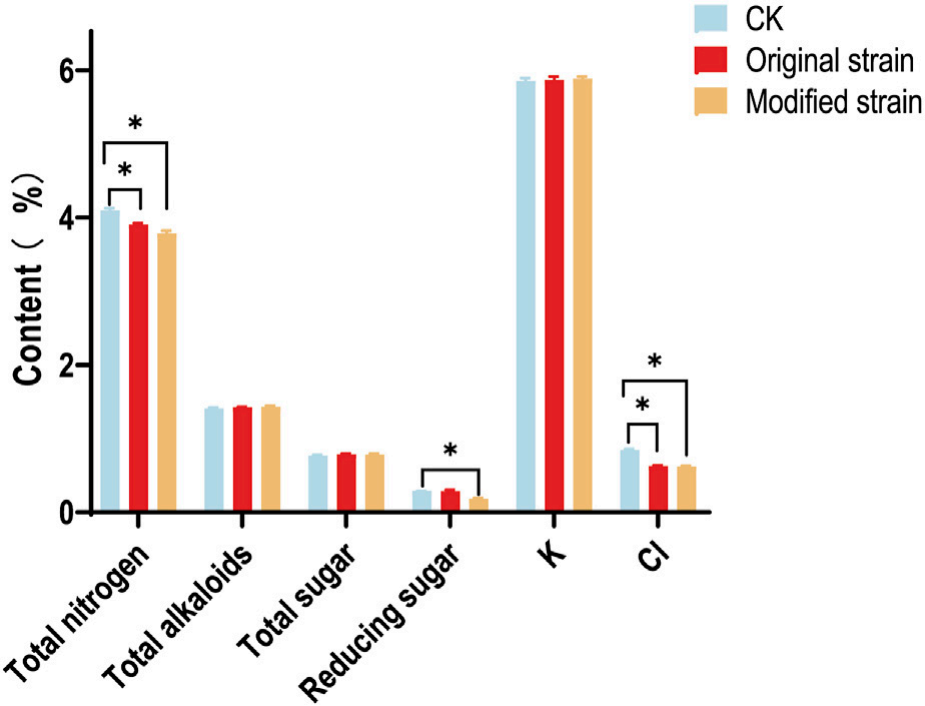
The chemical compositions of CTLs after original and modified Rh3 fermentation. CK represents control group. Asterisks indicate significant difference (Dunnett test, p < 0.05).

The chemical compositions of CTLs, including total nitrogen, total alkaloids, total sugar, reducing sugar, potassium (K), and chlorine (Cl), significantly influence their intrinsic quality. To assess whether the original or modified Rh3 causes changes in major chemical compositions, continuous flow analysis was performed. Results (Figure 5) showed that some major chemical compositions, such as total alkaloids, total sugar, and K, exhibited minimal variation among different groups. These three chemical compositions respectively influence the irritancy (Jia et al., 2023b; Yin et al., 2019), mainstream smoke (Yin et al., 2019), and combustibility (Wang et al., 2021) of CTLs. These results indicate that Rh3 does not cause negative consequences in these aspects.

It has been reported that the chlorine content is inversely related to the combustibility of CTLs (Hu et al., 2022b). Both the original and modified Rh3 groups showed a decrease in chlorine content compared with the control group. This phenomenon can be attributed to the volatilization of chlorinated compounds. Chlorine in CTLs may be absorbed by Rh3 strains or interact with the metabolic products of Rh3, forming certain volatile components, which leads to decreased chlorine content. This result indicates that both the original and modified Rh3 strains can improve the combustibility of CTLs through fermentation.

Reducing sugar plays a crucial role in cigar sensory quality (He et al., 2023). It can be converted to various aromatic substances during fermentation or pyrolysis reactions (Hu et al., 2022a; Talhout et al., 2006). Figure 5 also shows the changes in reducing sugar level after fermentation with modified Rh3. Interestingly, only the modified group exhibited a decrease in reducing sugar content. This may be attributed to the conversion of reducing sugar into flavor compounds, such as carotenoid degradation products, during fermentation.

More importantly, the total nitrogen content decreased following the fermentation of both original and modified Rh3 strains. Total nitrogen content is a key indicator of protein level (Boulos et al., 2020), and the protein in CTLs causes the emission of unpleasant odors during combustion. The reduction in total nitrogen content indicates that Rh3 may have the ability to promote protein degradation or amino acid deamination (Ghilardi et al., 2020; Jendresen et al., 2015), which could lead to a decrease in protein content in CTLs and improve the sensory quality of cigars. The above chemical compositions analysis results also demonstrated that Rh3 can improve the intrinsic quality of CTLs through fermentation. Furthermore, the introduction of CCD1 does not cause negative effect on this characteristic.

## 4 Discussion

The primary goal of cigar fermentation is to enhance aroma richness while reducing irritation in CTLs (Jia et al., 2023b). Through fermentation, Rh3 isolated from CTLs exhibits these desired effects. Based on Rh3’s carotenoid-producing property, we introduced CCD1 to enhance its flavor-producing capability through carotenoid degradation. This modification of the Rh3 strain enhanced aromatic compound production without altering its original characteristics (e.g., reduced total nitrogen content and chlorine content) during CTL fermentation.

Interestingly, the increased carotenoid degradation products observed in the culture medium (e.g., dihydro-beta-ionol, Figure 3A) differ from those generated during CTL fermentation (e.g., 3-hydroxy-5,6-epoxy-β-ionol, Figure 4B). This discrepancy is likely due to the minimal amount of inoculated Rh3, that is, only a small amount of dihydro-beta-ionol was introduced before fermentation, which has a negligible impact on the overall yield. The majority of carotenoid degradation products were generated during the 28-day fermentation period. In contrast to the defined nutrient composition and controlled environment of the culture medium, the complex substrate composition and environment during CTL fermentation may cause Rh3 to synthesize different carotenoids. For instance, environmental stress conditions have been shown to significantly influence the carotenoid biosynthetic profile of *Rhodotorula* species (Li et al., 2022). Additionally, previous studies have demonstrated that nicotine can induce the selective biosynthesis of lycopene and 3,4-didehydrolycopene, which are carotenoids not typically produced by *Rhodotorula* under standard conditions (Squina and Mercadante, 2005). Consequently, these distinct carotenoid profiles, when subjected to CCD1-mediated degradation in modified Rh3 during the CTL fermentation process, may result in different degradation product patterns between CTL fermentation and culture medium conditions.

Modified Rh3 exhibits distinct advantages over the exogenous addition of the CCD1 enzyme in CTL fermentation. The exogenous CCD1 enzyme is unable to penetrate the cell wall of *Rhodotorula*, limiting its ability to degrade intracellularly synthesized carotenoids. This limitation is similar to other carotenoid-degrading microorganisms and enzymes naturally present in CTLs. These biocatalysts can only degrade pre-existing extracellular carotenoids, which is a naturally constrained resource. In contrast, modified Rh3 remains viable throughout the fermentation process and continuously produces carotenoids that are subsequently degraded by endogenously expressed CCD1 to generate aroma compounds. Furthermore, unlike traditional engineered strains such as *E. coli* and *Y. lipolytica* (Basiony et al., 2022), the tobacco adaptability of modified Rh3 may facilitate a more harmonious fermentation process, potentially leading to the production of a more coordinated aroma profile that blends with the inherent tobacco flavors.

This study modified a non-traditional chassis microorganism isolated from CTLs and made a valuable attempt to utilize modified microorganisms for CTL fermentation. For future study, it is necessary to identify the specific types of carotenoids produced by Rh3 and to monitor the microbial community changes, functional gene expression and metabolite profiles through multi-omics methods to elucidate the mechanism of CTL fermentation by original and modified Rh3 strains. Considering the current limitations of applying genetic engineering techniques in tobacco production, utilizing modified Rh3 to enhance the flavor content of low-grade tobacco leaves for flavor extraction is worth exploring. Given the abundance of flavor compounds in tobacco leaves (Popova et al., 2019; Xu et al., 2013), this strategy could provide a viable option for upgrading tobacco by-products while meeting the increasing demand for natural flavoring substances in various industries.

## 5 Conclusion

In this study, a carotenoid-producing *Rhodotorula* strain Rh3 was isolated from CTLs and genetically engineered with the CCD1 gene to enhance its fermentation effects. After genetic engineering, the modified Rh3 exhibited a significant increase in carotenoid degradation product accumulation compared with the original Rh3 in culture medium (from 0.29 μg/mg to 15 μg/mg). Subsequent CTL fermentation experiments revealed that, the modified Rh3 outperformed both the control and the original strain. The modified strain showed a 65.9% increase in carotenoid degradation products compared with the control, while the original Rh3 achieved a 41.43% increase. These results demonstrate the enhanced fermentation effectiveness of the modified strain. Furthermore, analysis of the major chemical compositions revealed that the modified Rh3 maintained its ability to enhance the intrinsic chemical composition of CTLs. This study not only demonstrates the potential of genetically modified microorganisms in improving CTL quality but also provides a promising strategy for increasing the flavor content of low-grade tobacco leaves for flavor extraction.

## Data Availability

The raw data supporting the conclusions of this article will be made available by the authors, without undue reservation.
