# Supplementary material for: Introducing CCD1 into isolated Rhodotorula strain enhances flavor production and improves cigar fermentation
Source: Front Bioeng Biotechnol. 2024 Dec 3;12:1510075. doi: 10.3389/fbioe.2024.1510075 (PMC11650503; doi:10.3389/fbioe.2024.1510075)
Supplement: Supplementary file 1 [file DataSheet1.PDF]

Supplementary Material

1 Supplementary Data

Supplementary Data S1 Sequence of codon-optimized Osmanthus fragrans CCD1 gene

ATGGGCATGCAGGGCGAGGACGCGCAGCGCACGGGCAACATCGTCGCGGTCAAGCCGA
AGCCCTCGCAGGGCCTCACCAGCAAGGCGATCGACTGGCTCGAGTGGCTCTTCGTCAAG
ATGATGCACGACTCGAAGCAGCCGCTCCACTACCTCTCGGGCAACTTCGCGCCGGTCGA
CGAGACCCCGCCGCTCAAGGACCTCCCGGTCACGGGCCACCTCCCTGAGTGCCTCAACG
GCGAGTTCGTCCGCGTCGGCCCGAACCCGAAGTTCGCCTCGATCGCGGGCTACCACTGG
TTCGACGGCGACGGCATGATCCACGGCATGCGGATCAAGGACGGCAAGGCGACGTATG
TCTCGCGGTACGTCCAGACGTCGCGCCTGAAGCAGGAGGAGTTCTTCGGCCGCGCGATG
TTCATGAAGATCGGGGACCTGAAGGGCATGTTCGGGCTCCTCATGGTCAACATGCAGAT
GCTGCGCGCCAAGCTCAAGGTGCTGGACATCTCCTACGGCATCGGCACCGCGAACACGG
CCCTCGTCTACCACCACGGCAAGCTCCTCGCCCTCTCGGAGGCGGACAAGCCCTACGCC
ATCAAGGTCCTCGAGGACGGCGACCTCCAGACGATCGGCCTCCTGGACTACGATAAGCG
CCTCGCCCACAGCTTCACCGCACACCCTAAGGTCGACCCCTTCACGGGCGAGATGTTCA
CGTTCGGCTACTCGCACACGCCGCCTTACGTCACGTACCGGGTCATCTCGAAGGACGGA
GCGATGAACGACCCGGTCCCGATCACGGTCTCGGGCCCCATCATGATGCACGACTTCGC
CATCACCGAGAACTACGCGATCTTCATGGACCTCCCGCTCTACTTCAAGCCCAAGGAGA
TGGTGAAGGACAAGAAGTTCATCTTCTCGTTCGACGCCACGCAGAAGGCGCGCTTCGGC
ATCCTCCCGCGCTACGCGAAGAACGAGCTCCTGATCAAGTGGTTCGAGCTCCCGAACTG
CTTCATCTTCCACAACGCGAACGCGTGGGAGGAGGGCGACGAGGTCGTCCTCATCACGT
GCCGCCTCGAGAACCCTGACCTCGACATGGTCAACTCGACCGTCAAGGAGCGCCTAGAC
AACTTCAAGAACGAGCTCTACGAGATGCGCTTTAACCTCCAGAACGGCCTCGCCTCGCA
GAAGAAGCTCTCCGTGTCGGCGGTCGACTTCCCGCGCGTCAACGAGTCGTACACCACGC
GCAAGCAGCGCTACGTCTACGGCACGACGCTCGACAAGATCGCCAAGGTCACCGGCATC
ATCAAGTTCGACCTCCACGCGGAGCCCGAGACCGGCAAGGAGAAGCTCGAGCTCGGCG
GCAACGTCAAGGGCATCTTCGACCTCGGCCCCGGCCGCTTCGGCTCGGAGGCCGTGTTC
GTCCCCAGGCACCCAGGGATCACTAGCGAAGAGGATGACGGTTACCTCATCTTCTTTGTT
CACGATGAGAACACCGGCAAGTCGGCCGTCAACGTCATCGACGCCAAGACGATGTCGCC
GGACCCTGTCGCGGTCGTCGAGCTCCCGAAGCGCGTCCCCTACGGCTTCCACGCGTTCTT
CGTCACGGAGGACCAGCTCCAGGAGCAGGCGAAGGTCTGA

2 Supplementary Figure

Supplementary Figure S1

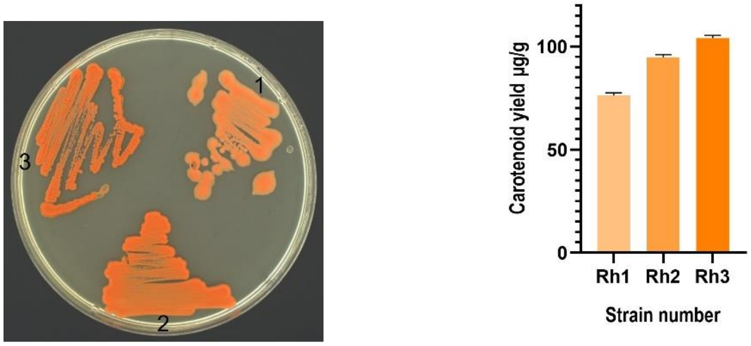

Supplementary Figure 1. the morphology and carotenoid yield of three isolated *Rhodotorula* strains.
